# Attention Deficit Hyperactivity Disorder Among Youth at Clinical High Risk of Psychosis

**DOI:** 10.1093/schizbullopen/sgae028

**Published:** 2024-11-06

**Authors:** Amy Braun, Lu Liu, Carrie E Bearden, Kristin S Cadenhead, Barbara A Cornblatt, Matcheri Keshavan, Daniel H Mathalon, Diana O Perkins, William S Stone, Ming T Tsuang, Elaine F Walker, Scott W Woods, Tyrone D Cannon, Jean Addington

**Affiliations:** Department of Psychiatry, Hotchkiss Brain Institute, University of Calgary, Calgary, AB, Canada; Department of Psychiatry, Hotchkiss Brain Institute, University of Calgary, Calgary, AB, Canada; Departments of Psychiatry and Biobehavioral Sciences and Psychology, Semel Institute for Neuroscience and Human Behavior, UCLA, Los Angeles, CA; Department of Psychiatry, UCSD, San Diego, CA; Department of Psychiatry, Zucker Hillside Hospital, Long Island, NY; Department of Psychiatry, Harvard Medical School at Beth Israel Deaconess Medical Center and Massachusetts Mental Health Center, Boston, MA; Department of Psychiatry, UCSF, and SFVA Medical Center, San Francisco, CA; Department of Psychiatry, University of North Carolina, Chapel Hill, NC; Department of Psychiatry, Harvard Medical School at Beth Israel Deaconess Medical Center and Massachusetts Mental Health Center, Boston, MA; Department of Psychiatry, UCSD, San Diego, CA; Institute of Genomic Medicine, University of California, La Jolla, CA; Departments of Psychology and Psychiatry, Emory University, Atlanta, GA; Department of Psychiatry, University of North Carolina, Chapel Hill, NC; Department of Psychiatry, Yale University, New Haven, CT; Department of Psychology, Yale University, New Haven, CT; Department of Psychiatry, Hotchkiss Brain Institute, University of Calgary, Calgary, AB, Canada

**Keywords:** psychosis, functioning, cognition, early intervention, transition, comorbidity

## Abstract

**Background:**

Attention Deficit Hyperactivity Disorder (ADHD) affects a significant proportion of the population and is associated with numerous adverse outcomes including lower educational attainment, occupational challenges, increased substance use, and various mental health issues including psychosis. This study examined the demographic, clinical, cognitive, social cognitive, and functional differences between youth at clinical high-risk (CHR) for psychosis with and without comorbid ADHD.

**Method:**

Data were drawn from the North American Prodrome Longitudinal Studies (NAPLS2 and NAPLS3), which included 764 and 710 CHR individuals, respectively. After applying exclusion criteria, the sample consisted of 271 CHR participants with ADHD and 1118 without ADHD. All data were examined cross-sectionally.

**Results:**

Compared with the non-ADHD group, the ADHD group was younger, had more difficulties with role functioning, premorbid functioning, and social cognition, were more likely to have a comorbid learning disorder, and reported less depression symptoms. There were no significant differences between the groups on positive or negative psychotic symptoms, transition rates, adverse events, or other comorbid disorders including substance use and depression.

**Discussion:**

Comorbid ADHD is likely not a significant predictor of transition to psychosis among CHR youth; however, those CHR with ADHD may experience symptoms at a younger age than those without and present with a distinct clinical profile.

## Introduction

A recent umbrella review estimated that rates of attention deficit hyperactivity disorder (ADHD) have been steadily increasing over the last several decades, with 7.2% of children and 2.5% of adults affected internationally.^1^ ADHD is a neurodevelopmental disorder characterized by difficulties regulating attention, disorganization, and/or hyperactivity/impulsivity, that is generally most effectively treated using psychostimulant medication.^2^ Presentation of ADHD differs between males and females, with males showing more hyperactivity symptoms and females displaying more issues with motor response inhibition, cognitive flexibility,^1^ and having less access to treatment due to more subtle symptom presentation.^3^ A diagnosis of ADHD is associated with several adverse outcomes including lower educational attainment, occupation challenges, increased rates of alcohol and tobacco consumption, accidental injuries and suicide, criminal activity, unwanted pregnancy, childhood trauma, chronic health conditions, and depression and anxiety.^1^ ADHD is also associated with deficits in social cognition^4^ and psychotic symptoms.^1,5^

Psychosis is a mental state characterized by a disconnection from reality and is a common feature of several mental disorders, including schizophrenia.^6^ Schizophrenia is characterized by positive symptoms such as delusions, hallucinations, and disorganized thoughts and behavior, as well as negative symptoms such as diminished emotional expression, social withdrawal, and impairments in cognition such as issues with attention and memory.^6^ Estimates suggest that schizophrenia affects ~1 in 300 individuals and contributes significantly to healthcare burden due to its severe, long-term consequences and poor functional outcomes.^6,7^ Those with schizophrenia often struggle to maintain steady employment, experience social difficulties, and face reduced life expectancy and high risk of suicide.^6,7^ Males tend to have more severe negative symptoms, such as poorer premorbid and current social functioning, increased substance use (including cannabis), an earlier age of onset, and generally a more chronic course with worse outcomes.^8^ Diagnosis of schizophrenia generally occurs in late adolescence or early adulthood, with individuals retrospectively reporting a period of functional and cognitive decline and subthreshold symptom onset preceding the first full-blown episode of psychosis.^9^

The pathway to schizophrenia is complex. However, Howes and Murray have published a seminal review integrating various models to explain the trajectory of schizophrenia.^10^ Their sociodevelopmental-cognitive model of psychosis combines the dopamine and neurodevelopmental hypotheses and postulates that genetic risk, subsequent developmental issues, and pre- and peri-natal hazards may sensitize the brain’s dopamine system.^10^ Later social adversities, such as childhood trauma, may then create biases in cognitive schema that lead individuals toward paranoid interpretations. The stress arising from these cognitive processes then feeds into further dopamine dysregulation and misattribution of stimuli, resulting in entrenched psychotic beliefs and hallucinations.^10^

The perspective of schizophrenia as a sociodevelopmental disorder featuring declining cognitive deficits raises questions about its potential shared etiology with ADHD. Howes and Murray^10^ report that there are several genes that are susceptible to schizophrenia which have also been implicated in other neurodevelopmental disorders. Evidence suggests that certain alleles may confer a polygenetic risk for both adult schizophrenia and ADHD^11^ and it has been found that those with ADHD may exhibit suppression of P50 auditory event-related potentials, similar to long-standing findings among those with schizophrenia spectrum disorders.^12^ Furthermore, the co-occurrence of ADHD and psychotic symptoms has been documented in the general population, and these two symptomologies may exacerbate each other.^13,14^ Stickley, Shirama and Sumiyoshi^13^ found that psychotic experiences are strongly associated with ADHD symptoms, and the rates of ADHD have been found to be much higher among youth with significant psychosis spectrum symptoms.^14^ Furthermore, individuals diagnosed with schizophrenia typically have higher rates of childhood and adult ADHD symptoms compared with healthy populations^15^ with the prevalence of comorbid ADHD possibly being more than twice that of the general population.^16^

Concerningly, the presence of ADHD may exacerbate certain negative outcomes among those with psychotic disorders as demonstrated by links to increased psychotic-like symptoms,^17^ poorer cognitive abilities, more suicide attempts,^18^ and poorer functioning.^19^ One study found that, among those with schizophrenia and ADHD, participants were more likely to be male and have a lower education level, more severe positive symptoms, and more severe cognitive deficits.^16^ It has been suggested that the co-occurrence of ADHD and psychosis could be attributable to the use of psychostimulants among those diagnosed with ADHD as children, although this evidence is inconclusive.^20^ However, one study did find that exposure to amphetamines and to atomoxetine was associated with an increased risk of newly diagnosed psychotic symptoms compared with nonexposure.^21^ Additionally, it has been found that those with psychosis who were exposed to stimulants had a younger age of psychosis onset,^22^ which is known to be associated with a poorer prognosis among those with psychotic disorders.^23^

While the evidence for ADHD symptoms as a precursor to early psychotic symptoms is unclear, a recent review and meta-analysis including 12 observational studies with participants in clinical and general populations did report a robust relationship between childhood ADHD and a later diagnosis of a psychotic disorder.^24^ This was the case even when controlling for sex and risk of bias and including only adjusted values.^24^ Although there was not enough data to investigate other covariates in the meta-analysis, several of the included studies controlled for psychiatric comorbidity, socioeconomic status, age, and maternal and infant health factors.^24^ For example, Bjorkenstam and colleagues^25^ found that ADHD was a risk factor for psychotic disorders even after adjustment for autism spectrum disorder (ASD) and substance use disorder. Additionally, this risk was greater for those taking ADHD medication.^25^

ADHD is typically diagnosed in childhood, before age 12, and, although psychotic disorders are typically diagnosed in late teens or early adulthood,^26^ recent research suggests that it may be possible to identify individuals who are considered to be at clinical high risk (CHR) of developing a psychotic disorder. Individuals at CHR of psychosis experience subthreshold psychotic symptoms, functional decline, and a range of adverse outcomes and experiences including higher rates of trauma, neuro- and socialcognitive deficits, stress, and cannabis use difficulties when compared with healthy controls.^27–31^ Heterogeneity of symptoms, comorbid diagnoses, outcomes, and trajectories appears to be the norm among CHR individuals.^32^ Thus, the study of transdiagnostic trajectories of CHR youth has increased, as developmental pathways to psychosis may vary.^32^ It is possible that neurodivergence may be linked to a specific trajectory or prognosis for CHR individuals. This young group of at-risk individuals provides a unique opportunity to study the possible links between the early development of psychosis and neurodevelopmental disorders such as ADHD.

CHR individuals are known to have higher rates of comorbid diagnoses, including ADHD, when compared with healthy controls,^33^ with estimated rates ranging from 17% to 50%,^33–35^ although the highest rate came from a small case study of 9 individuals.^35^ In one study, those at CHR had increased ADHD polygenic risk when compared with controls,^36^ and in another report, there was a greater risk of committing violence.^37^ Additionally, in the offspring of adults with schizophrenia, links between ADHD and poorer cognitive abilities have been observed.^38^ One small study, examining the differences between 14 CHR individuals with comorbid ADHD and 14 without, found no differences between the groups in attenuated psychotic symptoms, depression, sex, or age, but did find those with ADHD had significantly less negative symptoms.^39^

Given the cognitive and symptomatic links between ADHD and psychosis and the unique challenges that accompany ADHD, it is important to understand the unique experience of those CHR individuals who also have ADHD. The current study aims to examine potential differences in demographic, clinical, functional, cognitive, social cognitive, adverse experiences, and transition outcomes between CHR individuals who presented to the North American Prodrome Longitudinal Studies consortia (NAPLS2 and NAPLS3) with a current diagnosis of ADHD and those CHR individuals who did not. We hypothesize that those CHR participants diagnosed with ADHD may be more likely to be male and have more difficulties with cognition and social cognition, more severe symptoms, poorer functioning, report more childhood trauma, and be more likely to transition to psychosis.

## Methods

### Participants

All participants were pooled from two National Institute of Mental Health–funded studies: NAPLS2 and NAPLS3. These NAPLS studies were multisite longitudinal studies conducted at Emory University; Harvard University; the University of North Carolina Chapel Hill (UNC); Yale University; Zucker Hillside Hospital; the University of Calgary, and the University of California at Los Angeles (UCLA), at San Diego (UCSD), and at San Francisco (UCSF). Participants were recruited through referrals from health care providers, social services, educators, and self-referrals in response to community and academic presentations, mailouts, websites, and public service announcements. The most common source of referrals was self-referral (by participants, family, or friends), followed by referrals from mental health agencies either hospital- or community-based programs. Potential participants first underwent a telephone screen. Those who screened positive were invited to an in-person eligibility and consent evaluation. Further information on NAPLS2 and NAPLS3 recruitment and methods can be found elsewhere.^40,41^

All participants were between 12 and 30 years old and were included if they met the Criteria of Psychosis-Risk Syndromes (COPS) based on the Structured Interview for Psychosis-Risk Syndromes (SIPS).^42^ Exclusion criteria for NAPLS2 and NAPLS3 were meeting criteria for current or lifetime Axis I psychotic disorder, an IQ value of  <70, a history of a central nervous system disorder, or their diagnostic psychosis-risk symptoms were accounted for by another Axis 1 disorder. Participants were not excluded from NAPLS for having nonpsychotic DSM-5 disorders, such as substance use disorders, major depression, anxiety disorders, ASD, or learning disorders.

NAPLS2 and NAPLS3 had 764 and 710 CHR participants, respectively. For this project, participants were excluded if they were missing baseline DSM-5 criteria for ADHD (*n* = 85). Thus, 713 of the available participants were from NAPLS2 and 676 from NAPLS3. CHR participants with available baseline data describing the presence or absence of a DSM-5 ADHD diagnosis based on the Structured Clinical Interview for DSM-5 (SCID)^43^ were selected. Two hundred and seventy-one (19.5%) CHR participants met ADHD criteria and 1118 did not. Of those meeting ADHD criteria, 140 (19.6%) were from NAPLS2 and 131 (19.4%) were from NAPLS3. Of those who did not meet ADHD criteria, 573 were from NAPLS2 and 545 were from NAPLS3.

### Measures

All measures were chosen for their well-validated psychometric properties and have been previously validated for use among CHR individuals. Demographic information, including age, sex, race, living arrangement, school and work status, marital status, and information about prescription medications, were collected through self-report.

The presence of a DSM-5 diagnosis of major depressive disorder, anxiety disorders, alcohol, cannabis use disorder, and learning disorders was determined using the SCID.^43^ Diagnoses of ADHD and learning disorders were determined using DSM-5 criteria and relevant questions from the Kiddie-SADS.^44^

The COPS criteria, ie, attenuated psychotic symptoms syndrome (APSS); brief intermittent psychotic symptoms (BIPS); genetic risk and deterioration (GRD); and presence of psychosis criteria, were determined using the SIPS.^42^

### Clinical Symptoms

The severity of attenuated psychotic symptoms and negative symptoms was determined using the Scale of Psychosis-Risk Symptoms (SOPS).^42^ The Calgary Depression Scale for Schizophrenia (CDSS)^45^ was used to measure the level of depression. The CDSS has demonstrated good reliability and validity for use among CHR individuals.^46^ The Alcohol and Drug Use Scale (AUS/DUS) was used to assess the severity and frequency of alcohol, cannabis, and tobacco use.

### Functioning

Current functioning was assessed using the Global Functioning Scales: Social and Role (GF:S, GF:R).^47^ The GF:S scale focuses on social functioning, including family relationships, with an emphasis on relationships and interactions with peers. The GF:R scale focuses on school and work performance. Both scales are rated on a 10-point scale (10 = superior functioning, 1 = extreme dysfunction). Functioning before baseline was retrospectively collected using the Premorbid Adjustment Scale (PAS).^48^ The PAS is a 6-point scale measuring sociability, withdrawal, peer relationships, age-appropriate intimate relationships, and school achievement for three age periods: childhood (under age 11), early adolescence (12-15 years), and late adolescence (16-18 years). Higher scores indicate poorer premorbid adjustment.

### Neurocognition

The Wechsler Abbreviated Scale of Intelligence-Second Edition (WASI-II)^49^ was used to estimate IQ. Premorbid IQ was determined using the Wide Range Achievement Test-4 (WRAT-4).^50^

### Social Cognition

Social cognition measures were assessed only in NAPLS2. The Relationship Across Domains (RAD)^51^ test was used to measure social perception competence based on four relational models.^52^ Fifteen vignettes describing an interaction between two characters within the framework of one of the 4 relational models are presented to participants. Participants are then asked to rate “yes” or “no” to the likelihood of certain behaviors occurring using information from the vignettes.

The Awareness of Social Inference Test (TASIT)^53^ assesses comprehension of contextual clues and interpretation of social inference. The TASIT includes 16 short video scenes where actors have everyday conversations. The video scenes contain contextual clues about the speakers’ intentions, emotions, and beliefs. In half of the scenes, the primary speaker tells a lie, and in the other half, they use sarcasm. After the scene, participants respond to items about what the person in the scene was thinking, doing, feeling, and saying.

The computerized Penn Emotion Recognition Test (ER-40) and the Penn Emotion Differentiation Test (EDF-40)^54^ were conducted. The ER-40 measures facial affect recognition and includes 40 color photographs displaying human faces. The faces come in 5 conditions, expressing either anger, sadness, fear, happiness, or no emotion. The images are diverse in age, race, gender, and intensity of the emotion (with 4 high-intensity and 4 low-intensity expressions). The images are displayed in a random order and participants are asked to categorize the faces into one of the 5 options. The EDF-40 measures the ability to differentiate the intensity of emotion displayed in the same person. Twenty happy and 20 sad faces are presented for the participant to identify intensity, without having to identify the emotion itself.

### Adverse Experiences

Presence of childhood traumatic experiences including emotional neglect (individuals at home did not listen to problems, problems were ignored, or not being able to find any attention or support), psychological (being sworn at, receiving lesser treatment relative to siblings, unjustified punishment, or blackmailing), physical (being kicked, punched, or experiencing any other form of physical abuse), and sexual (touched sexually by anyone against one’s will, forced to touch anyone or were pressured into sexual contact against one’s will) abuse. Participant responses were coded as “yes” that one of these 4 traumatic experiences occurred, or “no” the experience did not occur. Bullying was coded as “yes,” or “no” it occurred. Abuse and bullying were determined using an adapted version of the Childhood Trauma and Abuse Scale.^55^

Perceived discrimination was measured using an adapted self-report measure.^56^ Perceived discrimination was only assessed in NAPLS2. Participants were asked if they had experienced discrimination due to skin color, ethnicity, gender, age, appearance, disability, sexual orientation, religion, or any other reason in the past year or in their lifetime. Participants answered either “yes” or “no.”

### Procedures

The NAPLS2 and 3 studies were approved by the Institutional Review Boards of all participating sites. Written informed consent, including parental consent for all minors, was provided for all participants. Raters at each site developed detailed vignettes based on SIPS ratings, which were presented at weekly conference calls for consensus decision on diagnosis and symptom ratings. Calls were attended by clinical raters from all sites and chaired by J. Addington and T. McGlashan. For any participants who may be considered to have made the transition to psychosis, the process was repeated using a similar vignette detailing the increase in symptoms. At these consensus calls, psychosis risk diagnosis and symptom ratings were confirmed.

All measures were conducted at baseline and the SIPS was readministered at the time of transition for those who experienced a transition to psychosis.

### Statistical Analysis

Individuals with baseline data and an ADHD diagnosis were selected for analysis. Differences between CHR participants with ADHD and those without were compared across demographic variables, SIPS and SCID diagnoses, trauma, symptoms, functioning, neurocognition, social cognition, perceived discrimination, and transition percentages. The comparison of transition times was conducted between individuals who transitioned with ADHD and those who transitioned without ADHD. All continuous variables were compared using independent samples *t*-tests and categorical variables were analyzed using chi-square tests. We assessed how the application of the Bonferroni correction influenced the significance of the comparisons. For participants with ADHD, the frequencies and percentages of each type of stimulant medication were calculated.

## Results

Two-hundred and seventy-one CHR participants were diagnosed with ADHD. Two-hundred and nineteen were most likely under 17 when they received their diagnosis, whereas 52 were 17 or older, and 37% were female in the younger group and 23% female in the older group.

Baseline differences in demographic variables between the ADHD and non-ADHD groups are presented in Table 1. CHR participants with ADHD were significantly younger, had fewer years of education, were more likely to be enrolled in school, and were more likely to identify as white. Compared with the non-ADHD group, there were significantly more males in the ADHD group and more participants with a first-degree relative with a psychotic disorder. The ADHD group was significantly less likely to be living independently and to identify as East Asian. There were no significant differences in employment or marital status. Of the ADHD participants, 26 (9.6%) were taking stimulant medication at baseline.

**Table 1.** Demographic Differences Between ADHD and Non-ADHD participants

	ADHD *n* = 271	Non-ADHD *n* = 1118	Test statistic	Significance value	Effect size
	*Mean (SD)*	*Mean (SD)*	*t*	*P*	*Cohens d*
Age	17.43 (3.99)	18.62 (4.19)	−4.23	<.001	−0.29
Years of education	10.56 (2.87)	11.57 (2.94)	−5.08	<.001	−0.34
	*Frequency (%)*	*Frequency (%)*	*X* ^*2*^	*P*	
Sex					
Male	178 (65.7)	593 (53.0)	14.12	<.001	
Female	93 (34.3)	525 (47.0)			
Racial^a^					
Indigenous Groups	6 (2.2)	18 (1.6)	21.20	.003	
Asian	12 (4.4)	116 (10.4)			
Black	36 (13.3)	148 (13.3)			
Central/South American	10 (3.7)	62 (5.6)			
West/Central Asia and Middle East	0 (0.0)	14 (1.3)			
White (European)	177 (65.3)	600 (53.8)			
Native Hawaiian or Pacific Islander	0 (0.0)	5 (0.4)			
Interracial	30 (11.1)	152 (13.6)			
Living arrangements^b^					
With Family/Spouse	227 (83.8)	866 (77.8)	8.64	.013	
Independent	36 (13.3)	228 (20.5)			
Other	8 (3.0)	19 (1.7)			
School enrollment Status					
Enrolled	236 (87.1)	906 (81.3)	5.11	.024	
Not enrolled	35 (12.9)	209 (18.7)			
Employment status					
Employed	60 (22.3)	322 (29.0)	4.83	.089	
Unemployed	208 (77.3)	787 (70.8)			
Full time parent	1 (0.4)	3 (0.3)			
First degree relative with a psychotic disorder	48 (17.8)	144 (12.9)	4.31	.038	
Marital status					
Single/never married	264 (97.4)	1060 (95.1)	2.86	.240	
Married/common law	6 (2.2)	45 (4.0)			
Was married^c^	1 (0.4)	10 (0.9)			

^a^Asian includes: Chinese, Japanese, Korean, Cambodian, Indonesian, Vietnamese, East Indian, Pakistani, Sri Lankan; West/Central Asia and Middle East includes: Egyptian, Lebanese, United Arab Emirates, Afghanistan, Iranian.

^b^Independent includes living alone, with roommates or in a group/boarding house; other includes living in a shelter or unknown.

^c^Was married includes separated or divorced.

Results for baseline differences in clinical variables between the ADHD and non-ADHD groups are presented in Table 2. There were no significant differences in any SIPS diagnoses (BIPS, APSS, or GRD). Individuals with ADHD were significantly more likely to be diagnosed with a DSM-5 learning disorder. Note that only NAPLS2 collected DSM learning disorder information. There were no significant differences for depressive disorder, anxiety disorders, cannabis, or alcohol use disorder. See the footnote in Table 2 for the breakdown of all DSM-5 disorders included in the cumulative anxiety and learning disorder variables.

**Table 2.** Clinical Differences Between ADHD and Non-ADHD Participants

	ADHD *n* = 271	Non-ADHD *n* = 1118	Test statistic	Significance value	Effect size
	Frequency (%)	Frequency (%)	*X* ^*2*^	*P*	
SIPS diagnoses					
BIPS	2 (0.7)	19 (1.7)	1.35	.245	
APSS	258 (95.2)	1068 (95.5)	0.05	.818	
GRD	21 (7.7)	89 (8.0)	0.01	.908	
SCID diagnoses^a^					
Depression	115 (42.4)	528 (47.2)	2.01	.156	
Anxiety	162 (59.8)	626 (56.0)	1.27	.259	
Cannabis use disorder	138 (50.9)	574 (51.3)	0.02	.901	
Alcohol use disorder	136 (50.2)	564 (50.4)	0.01	.938	
Substance use disorder	23 (8.5)	92 (8.2)	0.02	.890	
Learning disorder^*^	23 (16.4)	23 (4.0)	28.73	<.001^**^	
Bullying^b^	138 (50.9)	613 (54.8)	1.34	.247	
Abuse^c^					
None	154 (57.7)	531 (49.2)	8.22	.084	
1 type	45 (16.9)	201 (18.6)			
2 types	35 (13.1)	161 (14.9)			
3 types	27 (10.1)	133 (12.3)			
4 types	6 (2.2)	53 (4.9)			
	*Mean (SD)*	*Mean (SD)*	*t*	*P*	*Cohens d*
Symptoms					
SOPS positive total	12.68 (3.82)	12.26 (3.61)	1.69	.091	0.12
SOPS negative total	12.07 (6.13)	12.02 (6.21)	0.12	.904	0.01
CDSS	5.40 (4.60)	6.30 (4.66)	−2.82	.005^**^	−0.19
Substance use severity					
AUS/DUS tobacco	1.28 (0.55)	1.23 (0.49)	1.36	.175	0.10
AUS/DUS alcohol	1.36 (0.56)	1.42 (0.56)	−1.53	.126	−0.10
AUS/DUS cannabis	1.28 (0.56)	1.29 (0.56)	−0.18	.859	−0.01
Functioning					
GF: Social	6.33 (1.52)	6.31 (1.55)	0.23	.816	0.02
GF: Role	5.77 (2.04)	6.19 (2.22)	−2.86	.004^**^	−0.19
PAS Childhood	0.29 (0.18)	0.23 (0.16)	5.29	<.001^**^	0.36
PAS Early adolescence	0.35 (0.17)	0.30 (0.17)	4.32	<.001^**^	0.30
PAS Late adolescence	0.35 (0.19)	0.30 (0.18)	3.18	.002^**^	0.27
Cognition and Social Cognition					
WASI-2 IQ	102.93 (16.41)	105.09 (15.41)	−2.00	.046	−0.14
WRAT-4 Standard	105.72 (16.98)	107.71 (16.83)	−1.70	.090	−0.12
RAD total^*^	30.74 (5.83)	31.95 (5.11)	−2.30	.022	−0.23
TASIT total^*^	50.81 (6.66)	52.76 (5.78)	−3.29	.001^**^	−0.33
Facial affect recognition^*^	32.42 (3.45)	32.92 (3.38)	−1.52	.130	−0.15
Facial affect discrimination^*^	23.72 (6.53)	24.64 (5.71)	−1.47	.143	−0.16
Perceived Discrimination^*^					
Lifetime	2.43 (2.18)	2.86 (2.26)	−1.98	.048	−0.19
Past year	1.72 (1.88)	1.89 (1.93)	−0.94	.348	−0.09

^a^Depression indicates Major Depressive Disorder; anxiety includes obsessive compulsive disorder, posttraumatic stress disorder, panic disorder, agoraphobia, social anxiety disorder, specific phobia, and generalized anxiety disorder; learning disorder includes reading disorder, mathematics disorder, disorder of written expression, learning disorder, and expressive language disorder.

^b^Bullying includes both psychological and physical.

^c^Abuse includes any emotional neglect, psychological, physical, and sexual abuse.

^*^NAPLS2 sample only.

^**^Remained significant after Bonferroni correction.

*Note:* SIPS, Structured Interview for Psychosis-Risk Syndromes; BIPS, Brief Intermittent Psychotic Symptom State; APSS, Attenuated Positive Symptom State; GRD, Genetic Risk and Deterioration State; SCID, Structure Clinical Interview for DSM-5 Disorders; SOPS, Scale of Prodromal Symptoms; CDSS, Calgary Depression Scale for Schizophrenia; AUS/DUS, Alcohol/Drug Use Scale; GAF, Global Assessment of Functioning; GF: Social, Global Functioning: Social Scale; GF: Role, Global Functioning: Role Scale; PAS, Premorbid Adjustment Scale; WASI-II, Wechsler Abbreviated Scale of Intelligence—Second Edition; WRAT-4, Wide Range Achievement Test 4; RAD, Relationship Across Domains; TASIT, The Awareness of Social Inference Test.

Participants in the ADHD group had significantly lower levels of depression on the CDSS. There were no significant differences for SOPS positive or negative symptoms, or severity of tobacco, alcohol, or cannabis use. The ADHD group reported significantly poorer role functioning and premorbid adjustment for childhood, early, and late adolescence. However, there were no significant differences in social functioning.

Those with ADHD had significantly lower IQ scores on the WASI-II, but not on the WRAT-4. Social cognition was compared for the NAPLS2 participants only. It was found that those with ADHD reported significantly lower scores on the RAD and TASIT, with no differences for facial affect recognition or discrimination. However, for cognition and social cognition, only the TASIT comparison remained significant after the Bonferroni correction.

There were no significant differences between the 2 groups in reported experiences of bullying or in cumulative abuse experiences. Those with ADHD did report significantly less perceived discriminatory experiences in their lifetime. There was no significant difference in perceived discrimination in the past year, although this small significant difference did not hold after the Bonferroni correction.

The impact of the Bonferroni correction on the significance of these comparisons is indicated in Table 2. Learning disorder, CDSS, role functioning, premorbid adjustment for childhood, early, and late adolescence, and the TASIT remained significant after applying the Bonferroni correction.

There was a higher percentage of non-ADHD participants (*N* = 124, 11.09%) who transitioned to psychosis compared with ADHD individuals (*N* = 20, 7.38%). However, this was not significant (*P* = .07). There was no significant difference in time to transition between individuals who transitioned with and without ADHD (*P* = .43).

## Discussion

This study investigated ADHD among individuals at CHR for psychosis. Differences between those CHR individuals who had ADHD and those who did not in demographic characteristics, symptoms, functioning, other comorbidities, neuro- and social cognition, and transition were examined. Nearly 20% of the combined NAPLS-2 and NAPLS-3 samples met the criteria for ADHD, constituting the largest investigated sample of CHR individuals with ADHD to date. Previous CHR studies found rates of ADHD to be 25%^34^ and 50%.^35,39^ Our percentage is in line with the first study, although all previous studies have much smaller sample sizes, which could explain the high rate found in Mazzoni et al. and Ribolsi et al.,^35,39^ with only 5 and 14 ADHD participants, respectively. Additionally, just under 10% of our CHR participants with ADHD were taking stimulant medication at baseline.

This study found that CHR individuals with ADHD were younger, had fewer years of education, and were more likely to be enrolled in school. Additionally, they were more likely to be male (as hypothesized) and white compared with the non-ADHD group. While these results differ from the previous Ribolsi et al.^39^ study, which found no differences based on age or sex among CHR individuals, they are consistent with well-established demographic bias in ADHD diagnosis that generally, males are more likely to be diagnosed with ADHD than females^57^ and that females are likely underdiagnosed due to more subtle symptom presentation and misdiagnosis with emotional disorders.^3^

There were no significant differences in the specific psychosis risk diagnoses (BIPS, APSS, or GRD) between the 2 groups, although individuals with ADHD were more likely to have a learning disorder diagnosis. This is not surprising as around 30% of those with ADHD have a learning disorder.^58^ Our study did find that there was a higher prevalence of having a first-degree relative with a psychotic disorder in the ADHD group. Therefore, it is possible that CHR individuals with ADHD may be at risk for experiencing psychotic symptoms earlier in life.

Contrary to our hypothesis, the groups in this study did not differ on total positive or negative symptoms. The Ribolsi et al.^39^ study did report differences in negative symptoms, but each group only had 14 participants. Additionally, issues with executive functioning due to ADHD may explain the functioning results. While having ADHD did not seem to impact scores on social functioning, the ADHD group did show worse role functioning and premorbid adjustment across childhood and adolescence.

Of note was the finding that although the 2 groups did not differ on diagnoses of depression those CHR with ADHD had lower ratings on the CDSS than those without, though this represented a small clinical difference. This finding aligns with the Ribolsi et al. study^38^ where non-ADHD CHR participants had higher levels of depression. There is generally a relationship between ADHD and depression,^59^ although these results can vary and be mediated by many factors including emotion regulation, irritability, social difficulties, and adverse events.^60–63^

Finally, consistent with our hypothesis, participants with ADHD scored lower on WASI-II IQ and social cognitive measures assessing theory of mind and social perception skills, but not on facial affect recognition or discrimination tests. However, only the difference in the TASIT held after the Bonferroni correction. Individuals with ADHD tend to have impaired social cognition when compared with healthy controls.^64^

Contrary to our hypothesis, although a greater percentage of non-ADHD participants made the transition to psychosis, this result was not significant and the time to transition did not differ significantly between the groups. This is similar to a previous study comparing CHR with and without ASD, which found that while ASD exacerbated certain symptoms, it was not related to more severe positive or negative symptoms or risk of transition.^65^ Our transition findings may be somewhat surprising given the strong relationship found between ADHD and psychotic disorders previously.^24^ There is evidence that stimulant medication could increase the risk of transition to psychosis and could explain some of the relationship between ADHD diagnosis and psychotic illness.^66^ In our study, rates of stimulant medication use in the ADHD group were relatively low, which could account for the observed lower transition rate among the ADHD participants.

To the best of our knowledge, this is the first study to specifically examine the impact of comorbid ADHD on CHR individuals. Through utilizing data from the NAPLS consortium, we were able to analyze the differences between those CHR with and without ADHD in a large sample including participants from all over North America.

However, this was a secondary analysis of NAPLS2 and NAPLS3 data and as such, it has several limitations. First, due to the cross-sectional nature of the analysis, no causal interpretations can be drawn about the observed differences. Second, learning disorders, social cognition, and perceived discrimination data were only available for NAPLS2 participants. Third, since this was a secondary analysis of our NAPLS 2 and 3 data sets, there are potential limitations in our ADHD diagnosis, which was based on DSM/SCID diagnoses with added questions from the Kiddie-SADS. Our participants had an age range of 12-30 years, and ADHD criteria can differ depending on the age group. For adults, retrospective reports of childhood symptoms are required, whereas for those under 17, information is gathered from parents and teachers, in addition to current symptoms. Thus, assessment tools for ADHD in adults vs young adolescents and children differ. Although limited, we made an estimate of how many participants received their diagnosis before age 17 and how many after 17 years.

Given these limitations, in future studies, we would recommend that diagnoses of ADHD should be made using assessment tools that are specifically designed for children and young adolescents and also those specifically designed for older adolescents and adults. It is also important to report on the presence of hyperactivity symptoms and the age of the participant when they first received an ADHD diagnosis. More longitudinal data on the early cognitive development of those with ADHD and CHR, detailed timing of the onset of ADHD symptoms, and longer follow-up posttransition to psychosis would enhance our understanding of the similarities and differences in the trajectories of psychosis and ADHD.

In conclusion, a substantial minority of CHR youth presents with comorbid ADHD. These youth may have a distinct profile that is consistent with demographic biases reported among ADHD in general. Additionally, overlapping features between ADHD and CHR syndromes may point to some shared genetic etiology between the disorders. Although ADHD may not have implications for transition among CHR individuals, participants at CHR with ADHD may experience attenuated psychotic symptoms at a younger age than those without.

Since only 10% were receiving medication for ADHD, clinical implications include monitoring those with ADHD to determine if a stimulant medication might help. If not already receiving psychological interventions for coping with ADHD, this is potentially another useful intervention. Lastly, this study only examined baseline differences, and thus, future research could focus on longitudinal observations.
